# Ten Items to Find Them All: Shortening Scales for the Screening of Executive Function in Children With Attention Deficit/Hyperactivity Disorder

**DOI:** 10.62641/aep.v53i3.1883

**Published:** 2025-05-05

**Authors:** Hilario Blasco-Fontecilla, Marcos Bella-Fernández

**Affiliations:** ^1^Instituto de Transferencia e Investigación (ITEI), Universidad Internacional de La Rioja (UNIR), 26006 Logroño, Spain; ^2^Department of Psychiatry, Emooti, 28010 Madrid, Spain; ^3^Center of Biomedical Network Research on Mental Health (CIBERSAM), 28029 Madrid, Spain; ^4^Faculty of Health, Universidad Internacional de La Empresa (UNIE), 28015 Madrid, Spain; ^5^Department of Psychology, Universidad Pontificia de Comillas, 28108 Alcobendas, Spain

**Keywords:** test shortening, screening, executive dysfunction, ADHD

## Abstract

**Background:**

Attention Deficit/Hyperactivity Disorder (ADHD) is the most common neurodevelopmental disorder. The affectation of executive functions is stressed in the most recent research on ADHD, and many tests are used to assess it in ADHD, but they are usually time- and effort-consuming.

**Methods:**

From a database of a total of 222 children with ADHD, 59 of them suffering executive dysfunction, we took the most widely used tests for executive functions in ADHD (Behavior Rating Inventory of Executive Function (BRIEF), Swanson, Noland, and Pelham (SNAP)-IV, and Conners’ Parent Rating Scale (CPRS-R)) and applied several methods of test shortening: Item-total correlations from the Classical Test Theory, factor analysis and their subsequent factor loadings, elastic nets, and the Graded Response Model from the Item Response Theory models. Using the parameters or indicators provided by each of these methods, we selected the most discriminative items to develop a brief screening tool.

**Results:**

Our results show that different selection methods select different items. More importantly, we found that the shortened tests obtained this way are in general capable of discriminating between patients with and without ADHD. More precisely, all the shortened tests show high sensitivity, but relatively low specificity.

**Conclusions:**

Shortened tests can be used for screening purposes without having to administer full test versions.

## Introduction

Attention Deficit/Hyperactivity Disorder (ADHD) is the most common neurodevelopmental disorder worldwide [1]. Suffering ADHD has many negative effects in daily life, such as difficulty in adapting to a variety of tasks and jobs, additional risks of accidents and substance abuse, or weaker interpersonal relationships. ADHD diagnosis is clinical, being to date no definitive tests for assessing ADHD. The diagnosis of ADHD is made after taking a medical history and performing a clinical assessment, which typically includes interviews with the patient and their relatives, as well as psychological tests, all of which are examined by a psychiatrist or a neurologist. In the last years, emphasis has been placed on the affectation of executive function in ADHD patients [2, 3, 4], and some useful tests have been developed or adapted to help therapists correctly diagnose ADHD, such as the Behavior Rating Inventory for Executive Function (BRIEF) inventory [5] or Conners’ Continuous Performance Test (CPT) [6]. The time and effort required to complete the tests may be challenging for many ADHD potential patients, characterized by lower levels of intrinsic motivation and a greater ease of becoming bored [2]. For instance, CPT lasts 14 minutes, whereas the BRIEF test takes 10 to 15 minutes [7, 8], and these time lapses may be too exigent for ADHD children.

A variety of methods have been developed to develop brief versions of psychometric tests. These brief versions often consist of a selection of items from the test based on one or more criteria. Several approaches, including psychometric indicators [9] and some complex metaheuristics and algorithms [10, 11], have been proposed and used to make these item selections. Applying this diversity of item selection methods results in heterogeneous item sets with comparable properties, even from the same tests and the same database [9, 10]. In particular, for the case of ADHD diagnosis, to date, no brief, comprehensive tests have been developed. The most used tests take too much time, such as the BRIEF [5] or the CPT [6], or only contain items for inattention and hyperactivity, such as the Swanson, Noland, and Pelham (SNAP)-IV scale [12] or the Conners’ Parent Rating Scale (CPRS-R) [13].

The aim of this work is to develop and assess a brief test from the items of the most common screening tests for ADHD. We hypothesize that it is possible to reduce the number of items without losing significantly the capability to discriminate between patients with and without affectation on executive function. Our goal is to develop a short test, taking no more than 10 items, which preserves the original form’s discriminative power.

## Methods

### Participants

A total of 222 participants were recruited for the present study. The inclusion criteria were: being admitted to the Child and Adolescent Psychiatry Service at the Hospital Universitario Puerta de Hierro Majadahonda, Spain; being under 18 years; having received a diagnosis of ADHD; having been assessed on executive dysfunction. Ages varied from 3 to 17 (mean = 11.43 years, standard deviation = 3.49). All the participants were assessed by an expert child and adolescent psychiatrist. 59 patients from the sample were diagnosed with executive dysfunction. All the participants signed an adapted reported consent, and their parents or legal guardians signed a written informed consent, before participating in the study. The study was previously approved by the Hospital Universitario Puerta de Hierro Majadahonda Ethics Committee (code 15.17).

### Measurements

#### BRIEF

The BRIEF inventory [5] is a well-known assessment inventory for executive function. It has been widely used in samples of ADHD patients [14, 15]. It has adequate psychometric properties [5, 7, 8]. It consists of 86 items and takes up to 15 minutes to complete. For every item, 3 response options are available: never, sometimes, and often, depending on the frequency of the events described on the items. The inventory contains 8 clinical scales, converging in two second-order factors (behavioral regulation index and metacognition index) and a global score.

#### SNAP-IV

The SNAP-IV test [12] is a short test for assessing ADHD and its two subtypes, inattention and hyperactivity/impulsivity. It consists of 18 items scored on a 4-point Likert scale, 9 items to assess inattention, and 9 items to assess hyperactivity/impulsivity. It is one of the most widely used scales for ADHD assessment and it shows acceptable psychometric properties [16]. Its adaptation to Spanish [17] also has good psychometrical properties.

#### CPRS-R

The CPRS-R [13] is a useful, well-validated screening test for ADHD in children. It consists of 10 items with a 4-point Likert scale. CPRS-R has acceptable psychometrical properties and good validity for screening for ADHD patients [13, 18].

### Statistical Analysis

We performed a variety of methods proposed for shortening tests. In all cases, the predictors include the 86 items from the BRIEF inventory, the 18 items from the SPAN-IV test, and the 10 items from the CPRS-R test.

These methods are:

Item-test correlations [9]. This is one of the most common discrimination measures in Classical Test Theory. In our case, because we are taking the items from several tests, we cannot simply use the total score from one of them or correlate each item with the total score on the test that item belongs to. Instead, we calculated the sum of all items and correlated each item with that total score. We then selected the items with the largest correlations.

Factor loadings from Confirmatory Factor Analysis (CFA) [9, 10]. Kleka & Paluchowski [9] used an exploratory factor analysis and took factor loadings from it. In contrast, Schroeders *et al*. [10] used what they named “stepwise confirmatory factor analysis”: they iteratively performed a confirmatory factor analysis by removing the item with the smallest factor loading after each factor analysis until the desired number of items remained. We simply fitted a confirmatory factor model with all the items loading in one single factor and then selected the items with the largest loadings.

Regression through elastic nets [11]. Kleka & Paluchowski [9] and Artieda-Urrutia *et al*. [19], among others, used logistic or linear regression coefficients, but when predictors are highly correlated, such as items from the same inventory, classical regression models are less adequate. In these cases, elastic nets are a plausible alternative. It has been successfully used to select items from several tests for a brief screening tool [20]. For every trial, the sample was randomly split into two subsamples; the first subsample served to train the elastic net and obtain the parameter estimations, and the second subsample was devoted to regression coefficient estimations. We performed 100 iterations and summed the 100 regression coefficients for each item. Then, we selected the items with the largest sum of coefficients. Packages “glmnet” [21] and “caret” [22] were used for parameter estimation.

Graded Response Model [23] from Item Response Theory, using “mirt” package [24] for R. For each item, the Graded Response Model estimates as many parameters as the number of response options, plus a discriminability parameter, *a*. The higher the parameter *a*, the higher the item discriminability. We are taking the items with the largest *a* values.

We discarded using metaheuristics like Genetic Algorithms [10] and Ant Colony Optimization [25] due to the large sample sizes required.

Once the coefficients or parameters were obtained, we performed Receiver Operating Characteristic (ROC) curves to estimate the accuracy, sensitivity, and specificity of the items selected for each procedure to discriminate between patients with and without executive dysfunction. ROC curves are statistical tools widely used to assess the performance of a given classifier against a criterion, which is the variable that the classifier should predict; in this case, each one of the shortened tests obtained acts as a separate classifier, and the criterion is the diagnosis of executive dysfunction. ROC curves provide an estimation of the optimal classifier threshold and three measures of prediction quality: sensitivity, specificity, and accuracy. Applied to this case, the sensitivity is the ratio between the cases of executive dysfunction correctly detected by the classifier and the total number of cases in the sample (detected or undetected); a sensitivity of 0 means that the classifier does not detect any case of executive dysfunction, and a sensitivity of 1 means that the classifier detects all of them. Analogously, the specificity is the ratio between the non-patients of executive dysfunction correctly labeled and all the non-patients (either correctly labeled as such or falsely detected as patients); again, a specificity of 0 means that the classifier does not detect any non-patient, and a specificity of 1 means that the classifier correctly labels all non-patients as such. Last, the accuracy is the ratio between all the individuals correctly labeled and the whole sample. We used the “pROC” package [26] for R to calculate the ROC curves corresponding to each of the shortened tests.

Last, to assess structural validity, we performed parallel analyses on each of the four shortened tests to determine the number of factors to extract, and then we performed exploratory factor analysis with “promax” as the rotation method. We used the package “paran” [27] for the parallel analysis.

For all the statistical analyses described above, we used R, version 4.3.2. (The R Core Foundation, Vienna, Austria) [28].

## Results

**Fig. 1.**
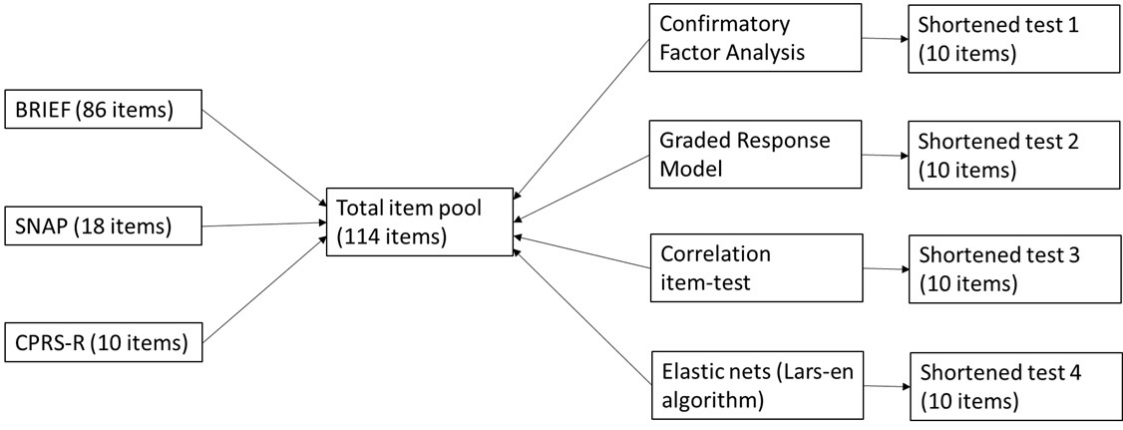
**Flowchart summarizing the process from the three original tests to the four shortened versions**. BRIEF, Behavior Rating Inventory of Executive Function; SNAP, Swanson, Noland, and Pelham; CPRS-R, Conners’ Parent Rating Scale.

Fig. 1 summarizes the method process from the original tests to the four shortened versions.

Table 1 shows the parameters or coefficients obtained from each procedure. Only one item, Item 7 from CPRS-R, was selected in the four procedures. Another item, Item 13 from SNAP-IV, was selected in three of the four procedures. The content of the selected items is described in the **Supplementary Material**.

**Table 1.** **Coefficients or parameters obtained**.

Item	Factor loading from CFA	Discrimination (a) parameters from GRM	Item-total correlations	Regression coefficients from elastic nets
SNAP 1	1.000	1.361	0.560	0.258
SNAP 2	1.113	1.671	**0.610**	0.093
SNAP 3	**1.146**	1.503	0.599	0.029
SNAP 4	1.123	1.456	0.542	0.086
SNAP 5	1.096	1.312	0.535	0.093
SNAP 6	**1.188**	1.510	**0.610**	0.002
SNAP 7	1.087	1.308	0.491	0.040
SNAP 8	1.117	1.654	**0.641**	0.038
SNAP 9	**1.256**	1.619	**0.620**	0.077
SNAP 10	0.816	1.077	0.454	0.119
SNAP 11	1.065	1.553	0.537	0.026
SNAP 12	1.067	1.488	0.556	0.045
SNAP 13	**1.144**	**2.044**	**0.651**	0.035
SNAP 14	0.881	1.205	0.487	0.006
SNAP 15	0.972	1.114	0.493	**0.337**
SNAP 16	**1.440**	1.446	0.412	0.121
SNAP 17	1.040	1.557	0.566	0.036
SNAP 18	**1.184**	**1.808**	0.514	0.182
CPRS-R 1	0.865	1.180	0.396	0.013
CPRS-R 2	0.527	0.752	0.363	0.051
CPRS-R 3	1.060	1.479	0.577	0.055
CPRS-R 4	0.898	1.421	0.504	0.141
CPRS-R 5	0.826	1.301	0.467	0.125
CPRS-R 6	**1.168**	**1.698**	0.604	0.199
CPRS-R 7	**1.220**	**1.751**	**0.634**	**0.901**
CPRS-R 8	0.685	1.000	0.450	0.026
CPRS-R 9	0.830	1.351	0.526	0.073
CPRS-R 10	0.864	1.401	0.499	0.019
BRIEF 1	0.385	0.742	0.399	0.224
BRIEF 2	0.752	1.350	0.517	0.033
BRIEF 3	0.361	0.419	0.243	0.125
BRIEF 4	0.491	0.901	0.409	0.306
BRIEF 5	0.625	1.044	0.279	0.030
BRIEF 6	0.542	0.984	0.455	0.143
BRIEF 7	0.400	0.852	0.406	0.064
BRIEF 8	0.466	0.801	0.374	0.125
BRIEF 9	0.816	1.448	0.555	0.015
BRIEF 10	0.771	1.398	0.562	0.026
BRIEF 11	0.837	1.423	0.515	0.022
BRIEF 12	0.456	0.788	0.422	0.215
BRIEF 13	0.407	0.849	0.369	0.077
BRIEF 14	0.693	1.492	0.526	0.073
BRIEF 15	0.597	0.868	0.401	0.156
BRIEF 16	0.482	0.759	0.371	0.038
BRIEF 17	0.735	**1.757**	0.559	0.039
BRIEF 18	0.651	1.189	0.438	0.022
BRIEF 19	0.737	1.627	0.549	0.085
BRIEF 20	0.221	0.573	0.252	0.229
BRIEF 21	0.607	1.411	0.521	0.041
BRIEF 22	0.759	1.147	0.479	0.192
BRIEF 23	0.459	0.770	0.404	**0.415**
BRIEF 24	0.839	1.594	0.582	0.097
BRIEF 25	0.476	1.059	0.454	0.014
BRIEF 26	0.342	0.756	0.352	0.036
BRIEF 27	**1.249**	**1.690**	0.490	0.242
BRIEF 28	0.672	1.201	0.490	0.203
BRIEF 29	0.309	0.569	0.246	0.308
BRIEF 30	0.327	0.538	0.288	0.036
BRIEF 31	0.862	0.749	0.361	0.069
BRIEF 32	0.734	1.281	0.505	0.049
BRIEF 33	0.645	1.058	0.461	0.118
BRIEF 34	0.737	1.491	0.565	0.172
BRIEF 35	0.703	1.416	0.526	0.015
BRIEF 36	0.646	1.502	0.515	0.031
BRIEF 37	0.915	**2.085**	**0.624**	0.035
BRIEF 38	0.790	1.485	0.572	0.160
BRIEF 39	0.374	0.675	0.336	**0.328**
BRIEF 40	0.608	1.142	0.474	**0.513**
BRIEF 41	0.836	1.656	0.604	0.044
BRIEF 42	0.850	**1.872**	**0.615**	0.023
BRIEF 43	0.685	1.472	0.582	0.095
BRIEF 44	0.685	1.560	0.566	0.068
BRIEF 45	0.605	1.247	0.516	0.038
BRIEF 46	0.776	1.326	0.521	0.078
BRIEF 47	0.783	1.451	0.545	0.142
BRIEF 48	0.726	1.001	0.297	0.008
BRIEF 49	0.674	1.098	0.483	0.026
BRIEF 50	0.759	1.030	0.398	0.046
BRIEF 51	0.886	1.420	0.579	0.052
BRIEF 52	0.661	1.255	0.501	0.066
BRIEF 53	0.796	1.378	0.505	0.041
BRIEF 54	0.478	1.173	0.471	0.083
BRIEF 55	0.686	1.516	0.594	0.013
BRIEF 56	0.645	1.318	0.460	0.013
BRIEF 57	0.753	1.222	0.522	0.260
BRIEF 58	**1.134**	1.482	0.459	0.068
BRIEF 59	0.590	1.206	0.483	0.131
BRIEF 60	0.831	1.463	0.527	**0.314**
BRIEF 61	0.504	0.722	0.348	0.026
BRIEF 62	0.435	1.069	0.415	**0.339**
BRIEF 63	0.708	1.488	0.534	0.009
BRIEF 64	0.476	1.017	0.451	0.137
BRIEF 65	0.887	**1.921**	**0.661**	0.028
BRIEF 66	0.710	1.345	0.533	0.064
BRIEF 67	0.633	1.053	0.474	0.055
BRIEF 68	0.701	1.221	0.525	**0.318**
BRIEF 69	0.618	0.960	0.233	0.089
BRIEF 70	0.433	0.925	0.419	0.086
BRIEF 71	0.219	0.253	0.201	0.126
BRIEF 72	0.456	0.702	0.351	0.194
BRIEF 73	0.724	1.513	0.539	0.037
BRIEF 74	0.740	1.437	0.507	0.091
BRIEF 75	0.677	1.208	0.517	0.276
BRIEF 76	0.647	0.820	0.373	0.048
BRIEF 77	0.878	1.606	0.587	0.041
BRIEF 78	0.781	1.399	0.511	0.080
BRIEF 79	0.866	**2.056**	**0.667**	0.079
BRIEF 80	0.751	1.398	0.556	0.064
BRIEF 81	0.581	1.053	0.463	0.112
BRIEF 82	0.747	1.434	0.577	0.199
BRIEF 83	0.789	1.475	0.588	0.214
BRIEF 84	0.699	1.385	0.559	0.043
BRIEF 85	0.783	1.235	0.575	**0.342**
BRIEF 86	0.826	1.232	0.531	**0.482**

The ten best values for each procedure are highlighted in bold. CFA, Confirmatory Factor Analysis; GRM, Graded Response Model.

Table 2 shows the accuracy, sensitivity, and specificity for each item selection procedure and the total item bank. In general, sensitivities are high, and specificities are low, which means that these tools are generally able to detect cases of executive dysfunction, but they also tend to falsely detect patients without it. Accuracy values, as global measures of classification performance, are low, likely because the sample has many more controls (patients without executive dysfunction) than patients, and thus the false positives overweight the correct positives in the global accuracy measure.

**Table 2.** **Discriminability measures of the items selected through each procedure**.

	Area under ROC curve	Accuracy	Sensitivity	Specificity
CFA	0.6214	0.460	0.922	0.277
GRM	0.6274	0.507	0.839	0.369
Item-total correlation	0.6254	0.522	0.763	0.423
Elastic nets	0.6142	0.516	0.777	0.407
All items	0.5854	0.482	0.830	0.356

ROC, Receiver Operating Characteristic.

We then divided the sample into two subsamples of patients with inattentive (n = 185) or mixed (n = 414) ADHD. We then also assessed the accuracy, sensitivity, and specificity of the shortened tests in the two subsamples. Table 3 shows the result of this differentiated assessment. In general, we can see that the shortened versions obtained through CFA, Graded Response Model(GRM), and item-test correlations show better performance in patients with inattentive ADHD than in patients with mixed ADHD. The shortened version obtained through CFA shows a large sensitivity in patients with inattentive ADHD and an acceptable specificity in patients with mixed ADHD. The shortened tests obtained through GRM and item-test correlation show acceptable sensitivities in patients with inattentive ADHD.

**Table 3.** **Discriminability measures of the shortened tests in patients with inattentive and mixed ADHD**.

		Area under ROC curve	Accuracy	Sensitivity	Specificity
Inattentive ADHD					
	CFA	0.681	0.578	0.917	0.400
	GRM	0.703	0.671	0.731	0.632
	Item-total correlation	0.675	0.630	0.731	0.574
	Elastic nets	0.574	0.575	0.577	0.574
Mixed ADHD					
	CFA	0.602	0.638	0.478	0.709
	GRM	0.600	0.605	0.543	0.634
	Item-total correlation	0.599	0.642	0.543	0.686
	Elastic nets	0.591	0.633	0.522	0.683

ADHD, Attention Deficit/Hyperactivity Disorder.

### Reliability and Content Validity

#### Confirmatory Factor Analysis

The items selected through factor loadings from CFA were: items 3, 6, 9, 13, 16, and 18 from SNAP-IV, items 6 and 7 from CPRS-R, and items 27 and 58 from BRIEF. The factor loadings selected ranged from 1.44 and 1.13. The internal consistency of the shortened test is high (alpha = 0.85, omega = 0.85).

The items refer to impulsivity (“He or she tends to talk excessively”), distractibility (“He or she forgets daily activities”, “He or she is easily distracted”), and difficulties in planning and persevering without external motivation (“He or she has difficulties to persevere in necessary actions to reach a certain goal, such as saving money to buy a special item or studying to get good grades”).

#### Graded Response Model From Item Response Theory

The items selected were: Items 17, 27, 37, 42, 65, and 79 from the BRIEF, items 13 and 18 from the SNAP test, and items 6 and 7 from the CPRS-R test. Their discriminability parameters ranged from 1.81 to 2.27. The internal consistency of the shortened test is high (alpha = 0.88, omega = 0.88).

Similarly to the CFA, the items selected through GRM refer to impulsivity and lack of inhibition, distractibility, and planning difficulties, but there is also one item referred to inability to detect negative reactions to his/her behavior.

#### Correlation Item-Test

The items selected were: items 37, 42, 65, and 79 from BRIEF, the items 2, 6, 8, 9, and 13 from SNAP test, and item 7 from CPRS-R. The item-total correlations of the selected items ranged from 0.667 to 0.610. The internal consistency of the shortened test is high (alpha = 0.89, omega = 0.89).

The items selected are also referred to impulsivity and lack of inhibition, distractibility, planning difficulties, and inability to detect negative reactions.

#### Elastic Net

The items selected were the item 15 from the SNAP-IV, the item 7 from the CPRS-R, and the items 23, 39, 40, 60, 62, 68, 85, and 86 from the BRIEF. The aggregated coefficients ranged from 0.901 to 0.314. The internal consistency of the shortened test is acceptable (alpha = 0.69, omega = 0.70).

The items selected refer to distractibility, planning difficulties, cognitive rigidity, emotional dysregulation, lack of inhibition, and impulsivity.

### Structural Validity

To assess the structural validity, we performed parallel analyses to extract the number of dimensions and exploratory factor analyses to obtain the factor loadings from each item. Tables 4,5,6,7 show the factor loadings for each shortened test; only factor loadings above 0.3 are included. The number of factors extracted from each shortened test is determined by the result from the respective parallel analyses.

**Table 4.** **Factor loadings for the shortened test from CFA**.

	Factor 1	Factor 2
SNAP 16	0.405	
SNAP 9		1.000
BRIEF 27	0.745	
CPRS-R 7	0.774	
SNAP 6	0.748	
SNAP 18	0.673	
CPRS-R 6	0.479	
SNAP 3	0.568	
SNAP 13	0.697	
BRIEF 58		0.831

**Table 5.** **Factor loadings for shortened tests from GRM**.

	Factor 1
BRIEF 37	0.733
BRIEF 79	0.616
SNAP 13	0.551
BRIEF 65	0.806
BRIEF 42	0.454
SNAP 18	0.692
BRIEF 17	0.726
CPRS-R 7	0.742
CPRS-R 6	0.536
BRIEF 27	0.723

**Table 6.** **Factor loadings for the shortened test from item-total correlations**.

	Factor 1	Factor 2
BRIEF 79		0.575
BRIEF 65		0.627
SNAP 13		0.872
SNAP 8	0.753	
CPRS-R 7	0.975	
BRIEF 37	0.662	
SNAP 9	0.721	
BRIEF 42		0.653
SNAP 6	0.633	
SNAP 2		0.914

**Table 7.** **Factor loadings for the shortened test from elastic nets**.

	Factor 1	Factor 2	Factor 3
CPRS-R 7			0.363
BRIEF 40	0.567		
BRIEF 86			0.525
BRIEF 23		0.744	
BRIEF 85	0.396		
BRIEF 62		0.603	
SNAP 15	0.685		
BRIEF 39	0.439		
BRIEF 68			0.399
BRIEF 60	0.323	0.347	

Factor 2 contains 2 items related to daily and time planification. Factor 1 includes items related to impulsivity, distractibility, and perseverance.

In this case, all the items load to a common factor including items of inattention and executive functions.

In this case, items from Factor 1 relate to distractibility and sustained attention, while items from Factor 2 are related to impulsivity and perseverance.

In this case, Factor 1 contains items related to talking too much and regarding closed topics, as well as one item pertaining to time estimation; Factor 2 contains items related to cognitive flexibility, emotional regulation, and detail orientation; and Factor 3 contains items pertaining to distractibility and difficulties to keep routines. In general, factor structures change in the shortened tests related to the original tests.

## Discussion

We used four procedures to shorten a battery of the most widely used tests to assess executive dysfunction in children with ADHD. Our results show that, in general terms, the four collections of items show acceptable psychometric and discriminant properties, despite containing substantially different items; only one item, item 7 from the CPRS-R test, is on the four item collections. In particular, the elastic nets tended to select different items than the three other procedures. This paradoxical effect of similar properties despite differences in selected items is consistent with other studies on test shortening [9].

The four item selection methods obtained generally large sensitivities (above or around 0.7) and low specificities. These results were particularly good in patients with inattentive ADHD, but none of these tests obtained good sensitivities or specificities in patients with mixed ADHD. In this context, these results mean that the four shortened tests have in general adequate properties to detect cases of executive dysfunction among patients with ADHD, especially with inattentive ADHD, leaving few cases undetected. However, they are also prone to falsely detect cases of executive dysfunction in patients who do not suffer from it. These results provide evidence of the adequacy of using these shortened tests as screening tools, but not as diagnostic tools. Rather, in the cases where any of these tests detect a possible case of executive dysfunction, a deeper assessment should be performed before diagnosing a patient with executive dysfunction.

Shortening tests usually has a negative impact on reliability. Shortened tests tend to have lower reliability than original tests [29]. In our case, the shortened versions obtained from CFA, GRM, and item-total correlations show large reliability coefficients, while the shortened version obtained through elastic nets has an acceptable internal consistency. This difference is not surprising: selecting the best item-total correlations is a good manner to optimize internal consistency [30], and the same logic might be applied to selecting the items with the largest factor loadings. On the contrary, GRM and elastic nets are based on discriminability. Despite this, GRM seems to outperform elastic nets in this regard.

Another common objection regarding shortened tests is the lack of validity that shortened versions suffer compared with the original tests [29, 31], and our case is not an exception. As suggested by several authors [29, 31, 32], we assessed the validity of the shortened versions in three ways: content validity, examining the theoretical content of the selected items, structure validity, assessing the factor structure of the shortened versions, and criterion validity in the sense of the shortened tests’ accuracy discriminating between individuals with and without executive dysfunction. We obtained substantial variations from the original contents and factor structure. The shortened tests only showed good sensitivity in detecting cases of executive dysfunction among patients with ADHD. Any other use for these shortened tests should be avoided without careful validity analysis.

The four procedures selected items which were related to impulsivity, lack of inhibition, and difficulties in planning and reaching long-term goals. GRM and item-total correlation also selected an item related to a lack of detection of negative reactions. The elastic nets included a wider variety of executive functions: apart from the features mentioned above, this method also selected items related to cognitive flexibility and emotional regulation. Recently, another study [33] used machine learning techniques to shorten the BRIEF-2 and found that Lasso algorithm (our elastic net) performed the best and selected items to predict ADHD. In our case, the goal was slightly different (to assess executive dysfunction in samples of patients already diagnosed with ADHD) and we fixed the number of items to be selected, whereas their study allowed a variable number of selected items.

The main limitation of this study is the potential use of the shortened tests obtained. Our goal was to obtain screening tests to detect executive dysfunction in children diagnosed with ADHD. Although this specific goal was achieved satisfactorily, especially in patients with inattentive ADHD, the tests obtained here showed that their usefulness beyond this application is scarce. In this regard, future research should cover the feasibility of these shortened test versions, focusing on completion times, and the experience from both patients and practitioners. Furthermore, our sample consisted of 222 patients with ADHD, 59 patients who also suffered executive dysfunction (cases), and 163 patients without executive dysfunction (controls). This relatively small sample did not allow us to use some meta-heuristic methods, such as Genetic Algorithms and Ant Colony Optimization, which could provide different shortened tests. Moreover, the sample size of this study highlights the need to generalize and replicate these findings in other samples of patients with ADHD.

## Conclusions

We obtained four shortened tests by applying four different shortening methods to the same database. The four shortened tests obtained in this work are adequate to be used for executive dysfunction screening purposes in the context of child psychiatry and psychology. Their usefulness as a screening tool for executive dysfunction was assessed through ROC curves. Content validity assessments reveal that the content loss due to the test shortening strongly discourages their use for other purposes than executive dysfunction screening in patients with ADHD. Further research and analyses are required to assess their usability beyond the scope of this work.

## Availability of Data and Materials

The datasets generated and/or analyzed during the current study are not publicly available due to confidentiality issues but are available from the corresponding author on reasonable request.

## Supplementary Material

Supplementary material associated with this article can be found, in the online version, at https://doi.org/10.62641/aep.v53i3.1883.
