# Supplementary material for: Ten Items to Find Them All: Shortening Scales for the Screening of Executive Function in Children With Attention Deficit/Hyperactivity Disorder
Source: Actas Esp Psiquiatr. 2025 May 5;53(3):546–56. doi: 10.62641/aep.v53i3.1883 (PMC12069905; doi:10.62641/aep.v53i3.1883)
Supplement: Supplementary file 1 [file ActEsp-53-3-546-556-s1.docx]

**Supplementary Material**

Items selected for each procedure

1. Confirmatory Factor Analysis

SNAP 16: Suele hablar en exceso. [He or she tends to talk excessively]

SNAP 9: Olvida actividades diarias. [He or she forgets daily activities]

BRIEF 27: Necesita la ayuda de un adulto para continuar con la tarea hasta terminarla. [He or she needs help from an adult to continue with tasks until ending them]

CPRS-R 7: Se distrae fácilmente, tiene escasa atención. [He or she is easily distracted and has low attention]

SNAP 6: En muchas ocasiones evita, rechaza o a regañadientes participa en tareas que le exigen un esfuerzo mental sostenido. [In many occasions, he or she avoids, rejects, or reluctantly takes part int tasks demanding excessive mental efforts]

SNAP 18: Suele interrumpir o entrometerse (por ejemplo, en conversaciones o juegos). [He or she tends to interrupt or meddles (for instance, in conversations or games)]

CPRS-R 6: No acaba las cosas que empieza. [He or she does not end the things which had started]

SNAP 3: A menudo parece que no escucha cuando se le habla directamente. [Often it looks like he or she does not listen when spoken directly]

SNAP 13: Suele tener dificultades para jugar o participar en actividades de forma ordenada o en silencio. [He or she tends to have difficulties to lay or take part in activities in a silent or ordered manner]

BRIEF 58: Tiene dificultad para mantener las acciones necesarias para alcanzar una meta (ahorrar dinero para un objeto especial, estudiar para obtener buenas calificaciones). [He or she tends to show difficulties to take the necessary steps to reach a goal (saving money for an especial object, study to get good marks…)

1. Graded response model

BRIEF 37: Tiene dificultades para terminar las tareas. [He or she shows difficulties to end tasks]

BRIEF 79: Se le dificulta reflexionar antes de actuar. [He or she gets difficult to think before act]

SNAP 13: Suele tener dificultades para jugar o participar en actividades de forma ordenada o en silencio. [He or she tends to have difficulties to lay or take part in activities in a silent or ordered manner]

BRIEF 65: Habla en el momento inadecuado. [He or she talks in inadequate moments]

BRIEF 42: Tiene dificultades para notar cuándo su comportamiento causa reacciones negativas. [He or she shows difficulties to notice when his or her behavior is causing negative reactions]

SNAP 18: Suele interrumpir o entrometerse (por ejemplo, en conversaciones o juegos). [He or she tends to interrupt or meddles (for instance, in conversations or games)]

BRIEF 17: Tiene dificultades para concentrarse en tareas o en el trabajo escolar, etc. [He or she shows difficulties to focus on tasks or school work]

CPRS-R 7: Se distrae fácilmente, tiene escasa atención. [He or she is easily distracted and has low attention]

CPRS-R 6: No acaba las cosas que empieza. [He or she does not end the things which had started]

BRIEF 27: Necesita la ayuda de un adulto para continuar con la tarea hasta terminarla. [He or she needs help from an adult to continue with tasks until ending them]

1. Correlation item-total

BRIEF 79: Se le dificulta reflexionar antes de actuar. [It is difficult to him or her to think before act]

BRIEF 65: Habla en el momento inadecuado. [He or she talks in inadequate moments]

SNAP 13: Suele tener dificultades para jugar o participar en actividades de forma ordenada o en silencio. [He or she tends to have difficulties to lay or take part in activities in a silent or ordered manner]

SNAP 8: Con frecuencia se distrae por estímulos externos. [He or she is easily distracted by external stimulation]

CPRS-R 7: Se distrae fácilmente, tiene escasa atención. [He or she is easily distracted and has low attention]

BRIEF 37: Tiene dificultades para terminar las tareas. [He or she shows difficulties to end tasks]

SNAP 9: Olvida actividades diarias. [He or she forgets daily activities]

BRIEF 42: Tiene dificultades para notar cuándo su comportamiento causa reacciones negativas. [He or she shows difficulties to notice when his or her behavior is causing negative reactions]

SNAP 6: En muchas ocasiones evita, rechaza o a regañadientes participa en tareas que le exigen un esfuerzo mental sostenido. [In many occasions, he or she avoids, rejects, or reluctantly takes part int tasks demanding excessive mental efforts]

SNAP 2: Con frecuencia tiene dificultad para permanecer atento en juegos o tareas. [He or she shows frequently difficulties to keep focused on games or tasks]

1. Lars-en

CPRS-R 7: Se distrae fácilmente, tiene escasa atención. [He or she is easily distracted and has low attention]

BRIEF 40: Subestima el tiempo necesario para acabar tareas. [He or she underestimates the time required to end tasks]

BRIEF 86: Tiene problemas para seguir la rutina matutina, estar listo para ir a la escuela, etc. [He or she has problems to follow morning routines, to be ready for school, etc.]

BRIEF 23: Se resiste al cambio de rutina, comida, lugares, etc. [He or she shows resistance to change routines, food, places, etc.]

BRIEF 85: Dice las mismas cosas una y otra vez. [He or she says the same things time after time]

BRIEF 62: Sus arranques de enfado o llanto son intensos, pero finalizan de repente. [His or her rage episodes are intense, but they end suddenly]

SNAP 15: Suele hablar en exceso. [He or she tend to talk too much]

BRIEF 39: Piensa demasiado sobre el mismo tema. [He or she overthinks about the same topic]

BRIEF 68: Deja sus pertenencias en dondequiera que vaya. [He or she leaves his or her belongings wherever he or she goes]

BRIEF 60: Su trabajo es descuidado. [His or her work is careless]
